# *Alternaria *and *Cladosporium *Fungal Allergen Epitopes are Denatured by Sodium Hypochlorite

**DOI:** 10.1097/WOX.0b013e3181c4c98f

**Published:** 2009-12-15

**Authors:** Charles Barnes, Freddy Pacheco, Minati Dhar, Jay Portnoy

**Affiliations:** 1 Section of Allergy/Immunology, The Children's Mercy Hospital, Kansas City, MO

**Keywords:** allergen, fungi, hypochlorite, *Alternaria*, *Cladosporium*, denature, human IgE

## Abstract

**Background:**

Fungal allergens are ubiquitous; however, little progress has been made understanding fungal allergenic material removal from indoor environments.

**Purpose:**

We investigated removal of environmental allergenic material derived from *Alternaria *and *Cladosporium *using sodium hypochlorite in vivo and in vitro.

**Methods:**

Freeze dried allergen extract from *Alternaria alternata *and *Cladosporium herbarum *was treated with hypochlorite concentrations of 322, 88, 38, 16, 3, 0.3, and 0 mM, respectively. Remaining native allergenic material was quantified using enzyme immunoassay and remaining viable fungal material was evaluated.

**Results:**

The results of treating *Alternaria *or *Cladosporium *extract with sodium hypochlorite are immediate and obvious. Concentrations greater than 100 mM remove color and concentrations between 100 and 38 mM partially uncolored the extract. Immunoassay for total antigenic and allergenic material remaining after treatment with sodium hypochlorite including 2 concentrations recommended for killing fungus confirmed a general destruction of antigenic and allergenic material at concentrations of 38 mM or greater.

**Conclusions:**

This work confirms the ability of solutions of sodium hypochlorite to denature fungal allergenic material from common outdoor and indoor fungi *A. alternata *and *C. herbarum*. Destruction of recognized antigenic and allergenic epitopes occurs at hypochlorite concentrations commonly used for household cleaning.

## 

Epidemiological studies have suggested that regular house-hold sodium hypochlorite use may have some benefit for persons suffering from allergies and asthma [1]. It has also been demonstrated that repeated treatment with low level sodium hypochlorite reduced the amount of dust borne environmental allergens [2,3]. Several authors have indicated that cedar pollen, cat, and mouse allergen can be successfully denatured by exposure to relatively low level hypochlorite [4-6]. In addition, it has been shown that fungal allergen containing material from *Aspergillus fumigatus *can be destroyed by exposure to sodium hypochlorite and this bleach exposure reduced the allergen content of *Aspergillus *extract below the detection limit of skin prick testing [7]. However, it has yet to be determined if hypochlorite treatment is sufficient to denature pigmented outdoor environmental fungi considered to be more resistant to environmental factors.

Fungal allergens are ubiquitous both indoors and outdoors; to date, the scientific world has made very little progress understanding factors related to the appearance and removal of this allergenic material in biologic samples. Allergens from fungi of the genus *Alternaria *are known to be associated with significant human allergy and are probably the most significant fungal allergens [8-10]. However, as far as total biomass is concerned, the fungal genus *Cladosporium *is probably the most abundant fungi in both the indoor and outdoor environment [11-13]. Therefore, we chose to investigate the fate of allergenic material derived from these 2 fungi both in vivo and in vitro when exposed to varying concentrations of sodium hypochlorite including 2 concentrations commonly used for household cleaning.

## Materials and Methods

### Materials

All studies involving human sera were performed after obtaining approval from the pediatrics institutional review board of the Children's Mercy Hospital. Polystyrene microtiter plates (immulon-2) were obtained from Dynatech (Chantilly, VA). Strains of *Alternaria alternata *(96154) and *Cladosporium herbarum *(6506) were obtained from ATCC (Manassas, VA) and allergen containing extract from the same strains was purchased from Greer Laboratories (Lenoir, NC). Antisera raised in rabbits injected with these same fungal strains were also obtained from Greer Laboratories. Sodium hypochlorite (Clorox) was obtained from a local store (Wall Mart, Lees Summit, MO) and was standardized for available chlorine by a colorimetric method [14]. Reagents and methods used for biotinylation were obtained from Pierce Chemical Company (Rockford, IL). Other conjugated antisera and related substrates for enzyme immunoassay were obtained from Sigma (St. Louis, MO). Water used was deionized and distilled unless otherwise noted.

### Antigen Denaturing Treatment

Freeze dried antigen containing material extracted at a 1:10 wt/vol ratio from *A. alternata *(ATCC 96154) and *C*. *herbarum *(ATCC 6506) was treated with freshly prepared hypochlorite solution at molar ratios (chlorine to antigen assuming antigen molecular weight of 30 kD) of 10,000, 2500 (equivalent to a 1:10 dilution of commercial hypochlorite in water), 1175 (equivalent to 3/4 cup commercial hypochlorite in 1 gallon of water), 500, 100, 10, and 0. This corresponds to hypochlorite millimolar (mM) concentrations of 322, 88, 38, 16, 3, 0.3, and 0, respectively. The antigen and hypochlorite mixture was allowed to incubate at room temperature for 10 minutes and then the hypochlorite was neutralized by adding an equivalent amount of sodium thiosulfate. In addition, the neutralized solution was made 1% in bovine serum albumin to stabilize the remaining antigen. Denatured, neutralized, and stabilized antigen solutions were stored at -20°C.

### Inhibition Immunoassay for Antigen Content

For inhibition immunoassays to determine the remaining antigen content of treated extract, microtiter plates were coated by dissolving native antigen at a concentration of 50 *μ*g dry weight/mL for both *Alternaria *and *Cladosporium *in a 0.15 M bicarbonate buffer (pH 9.6) (coating buffer) and adding 100 *μ*L of solution per well of a 96 well microtiter plate. The plates were then incubated at 4°C overnight. Optimal adsorption concentrations were determined empirically for each antigen antibody pairing.

Thus, plates coated with antigen extract were blocked with coating buffer containing 0.5% gelatin for 1 hour at 37°C and subsequently washed with 0.1 molar phosphate buffer (pH 7.4) containing 0.05% Tween 20 nonionic detergent (PT buffer) to remove nonadsorbed material. Next, 200 *μ*L/well of diluted antibody containing sera produced in rabbits along with preparations containing unknown amounts of fungal allergen material remaining after the denaturation process were added to the appropriate wells. The assay was standardized using antigen preparations of known protein content obtained commercially. Unknown antigen preparations were started at a 10-fold dilution and serially diluted 2-fold down the plate for 3, 2-fold dilutions. As a positive control to insure that residual chlorine or thiosulfate did not inhibit the assay, adjacent wells containing identical denatured antigen preparations were run with native antigen material added back to the assay. The wells were incubated at least 2 hours to come to equilibrium. After the equilibrium incubation, plates were washed with PT buffer and 100 *μ*L/well of 1:1000 alkaline phosphatase conjugated goat antirabbit IgG was added. After at least 1 hour incubation at 37°C, plates were washed with PT buffer and distilled water. After addition of 100 *μ*L of 1 mg/mL p-nitrophenyl phosphate, the color was allowed to develop until an internal standard read 1.0 absorbency units as measured at OD405 with a Dynatech MR7000 plate reader.

### Sandwich ELISA

For direct immunoassay of antigen content a series of preliminary experiments was performed to determine the optimum concentration of coating antibody and biotinylated detecting antibody for the assay. To perform the sandwich immunoassay, microtiter plates were coated with 5 *μ*g/well anti-*Alternaria *or 4.5 *μ*g/well anti-*Cladosporium *antibody in coating buffer and incubated overnight at 4°C. Coated plates were subsequently washed and blocked with 0.5% gelatin (BioRad, Hercules, CA) dissolved in coating buffer for 1 hour at 37°C to reduce background. Preparations containing known amounts of *Alternaria *or *Caldosporium *antigen obtained commercially or samples containing unknown amounts of fungal allergen material remaining after treatment with hypochlorite were added to the appropriate wells. Unknown antigen preparations were started at a 10-fold dilution and serially diluted 2-fold down the plate for 3, 2-fold dilutions. As a positive control to insure that residual chlorine or thiosulfate did not inhibit the assay, adjacent wells containing identical denatured antigen preparations were run with native antigen material added back to the assay. The wells were incubated at least 2 hours and washed with PT buffer. After washing, the plates were incubated with biotinylated anti-*Alternaria *or anti-*Cladosporium *antibody for 2 hours. After at least 1 hour incubation at 37°C with avidin alkaline phosphatase, plates were washed with PT buffer and distilled water. After addition of 100 *μ*L of 1 mg/mL p-nitrophenyl phosphate, the color was allowed to develop until an internal standard read 1.0 absorbency units as measured at OD405 with a Dynatech MR7000 plate reader.

### Antibody Biotinylation

Secondary antibodies used for the sandwich immunoassay were labeled with biotin. Two milligrams of lyophylized rabbit sera containing anti-*Alternaria *or anti-*Cladosporium *antibody was dissolved in 200 *μ*L of 0.05 M bicarbonate buffer (pH 7.5) and incubated at 4°C for 1 hour with 3 *μ*L of NHS-biotin (Pierce). At the end of this time, the sera was dialyzed at 4°C overnight against 0.1 M tris buffered saline and stored at -20°C in 50% glycerol at a protein concentration of 5 mg/ml. The activities of biotinylated antibodies were confirmed by direct ELISA using 1:1000 avidin-alkaline phosphatase (Sigma).

### Human IgE ELISA

For determination of allergenic material remaining after hypochchlorite treatment a second sandwich immunoassay was conducted in which IgE containing human sera was used as the detecting antibody. For this experiment, ELISA plates (immulon-2, Dynatech) were coated overnight with anti-*Alternaria *or anti-*Cladosporium *antibody in coating buffer as described above. Coated plates were blocked with 0.5% gelatin (BioRad) for 1 hour at 37°C. Serial dilutions of standard *Alternaria *or *Cladosporium *antigen extract or extract treated with varying concentrations of hypochlorite were added to appropriate wells and allowed to adsorb for 2 hours. As before a positive control to insure that residual chlorine or thiosulfate did not inhibit the assay was employed in that adjacent wells containing identical denatured antigen preparations were run with native antigen material added back to the assay. After washing, an aliquot of human serum from *Alternaria *or *Cladosporium *sensitive individuals previously selected for high IgE content was diluted 1:10 in PTA and placed into each well and incubated for 3 hours. After appropriate washing, human IgE directed alkaline phosphatase conjugated antibody made in goat and adsorbed against human IgG, IgA, and IgM (Sigma) diluted 1 to 1000 was then applied to the ELISA plate at 100 *μ*L/well in PTA. After 1 hour incubation, plates were washed with PTA and water. Alkaline phosphatase substrate was added as above and plates were allowed to develop until an internal standard read 1.0 absorbency units as measured at OD405 using a Dynatech MR7000 (Dynatech).

### Growth of Fungal Material on Drywall and Treatment with Hypochlorite

Squares of drywall 3 inches on each side were placed in large glass petri dishes, autoclaved and allowed to dry. Strains of *A. alternata *(96154) and *C. herbarum *(6506) were grown on MEA agar plates until colonies were confluent. Spores were then scraped from the surface of the plates and a suspension of spores containing greater than 1 million spores per milliliter was prepared. The squares of drywall were inoculated using cotton tipped applicators and aseptic technique. The squares were replaced into the dishes with 50 mL of sterile water. One piece of drywall was not inoculated but received 50 mL water to serve as a control. Squares were allowed to incubate in a humid chamber at room temperature for 3 weeks by which time heavy fungal growth was evident on all but the control square. At the end of 3 weeks, the squares were sprayed with solutions of sodium hypochlorite in sterile water at concentrations of 322, 81, 38, 16, and 0 mM, respectively. Each square received 10 actuations of the spray bottle. The bleach exposed drywall was allowed to incubate for 10 minutes and samples were taken from each square to test for fungal viability. The fungal material on the surface was then scraped off and extracted in 2 mL of PBS at room temperature for 2 hours. After 2 hours, solid material was removed by centrifugation and the extracts were tested for the presence of allergenic material.

### Test for Fungal Viability

To test for viability of the surface fungal growth after spraying with hypochlorite samples of the drywall surfaces were taken 10 minutes after spraying using sterile cotton tipped applicators. This applicator was then wiped evenly on the surface of a fresh MEA agar plate using aseptic technique. These plates were allowed to incubate at room temperature for 5 days when fungal colonies present were estimated.

## Results

**Figure 1:**
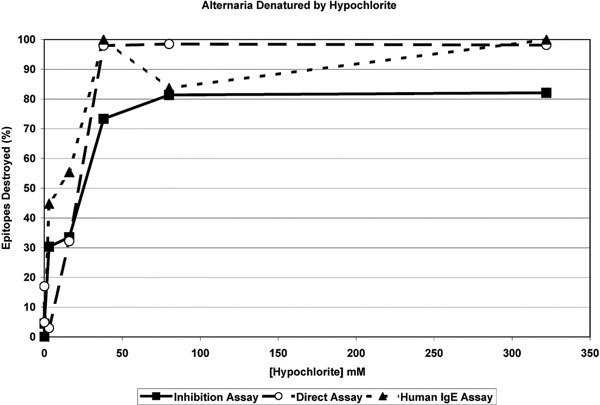
**Describes the percent of immunologically recognized epitopes remaining in a solution of *A. alternata *antigen material after treatment with increasingly dilute concentrations of sodium hypochlorite**. Three different assay methods are depicted including an inhibition immunoassay for total antigen epitopes, direct immunoassay for total epitopes, and direct immunoassay for allergenic epitopes. The figure indicates nearly all epitopes are destroyed by hypochlorite concentrations of 38 mM or greater.

Results of the determination of total antigenic material from *Alternaria *extract remaining in the native state as recognized by rabbit antibodies in inhibition and sandwich immunoassays and of total allergenic material recognized by human IgE in sandwich immunoassay are shown in Figure 1. The extracts were treated with sequential dilutions of sodium hypochlorite including 2 concentrations recommended by the manufacturer for killing fungus. Individual experiments representing antigen detected by inhibition immunoassay and direct immunoassay using rabbit antisera and allergen detected by direct immunoassay using IgE containing human sera previously determined to contain *Alternaria *specific IgE.

**Figure 2:**
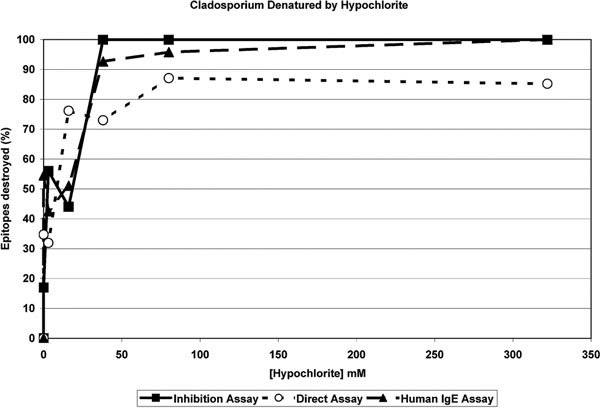
**Describes the percent of immunologically recognized epitopes remaining in a solution of *C. herbarum *antigen material after treatment with increasingly dilute concentrations of sodium hypochlorite**. Three different assay methods are depicted including an inhibition immunoassay for total antigen epitopes (solid squares), a direct immunoassay for total epitopes (open circles) and a direct immunoassay for allergenic epitopes (solid triangles). The figure indicates nearly complete epitope destruction by hypochlorite concentrations of 38 mM or greater.

Results of similar immunoassays for determinations of total antigenic and allergenic material from *Cladosporium *extract treated with dilutions of sodium hypochlorite are shown in Figure 2. Individual experiments representing antigen detected by inhibition immunoassay and sandwich immunoassay using rabbit antisera and allergen detected by sandwich immunoassay using human sera previously determined to contain IgE directed against *Cladosporium *are represented.

**Figure 3:**
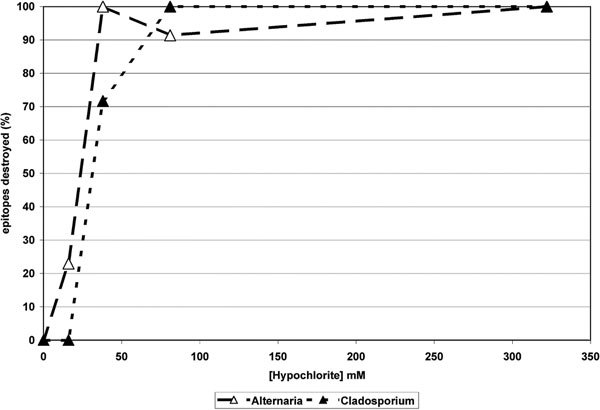
**Indicates the percent of epitopes remaining on an aequous extract of *A. alternata *fungal material (closed triangles) and *C. herbarum *fungal material (open triangles) grown on commercial drywall after treatment with dilute concentrations of sodium hypochlorite**. The figure indicates nearly complete epitope destruction by hypochlorite concentrations of 88 mM or greater.

Results for total *Alternaria *and *Cladosporium *allergenic material remaining in fungal matter removed from hypochlorite treated drywall are depicted in Figure 3. The fungi were grown on the previously sterilized drywall material for 3 weeks before treatment with differing concentrations of hypochlorite. The results provide an indication of native material recognized by the specific human IgE sandwich immunoassay used previously remaining after treatment with dilute solutions of hypochlorite.

The results of tests for viability of surface *Alternaria *growth on drywall after spraying with hypochlorite indicated that after 5 days there were no *Alternaria *colonies detected in surface drywall samples treated with 322, 81, and 38 mM hypochlorite. The surface drywall sample treated with 16 mM hypochlorite contained 9 *Alternaria *colonies and the *Alternaria *control drywall sample contained more than 4000 colonies. The results of tests for viability of surface *Cladosporium *growth after spraying with hypochlorite indicated that after 5 days there were no *Cladosporium *colonies detected from surface drywall samples treated with 322 and 81 mM hypochlorite. The surface drywall sample treated with 38 mM hypochlorite contained 34 *Cladosporium *colonies and the surface drywall sample treated with 16 mM hypochlorite contained 230 *Cladosporium *colonies. The *Cladosporium *control drywall sample contained more than 4000 colonies.

## Discussion

Sodium hypochlorite is the salt of hypochlorous acid that is one of the species present when hydrochloric acid is diluted in water. Strong concentrations of hydrochloric acid (6 M) are routinely employed in amino acid analysis [15] to completely destroy proteins breaking them into their individual amino acid constituents. Lower levels of hypochlorous acid produced when sodium hypochlorite is dissolved in water at millimolar concentrations are known to be strong sulfhydryl inhibitors inactivating enzymes containing sulfhydryl groups at concentrations less than 10 mM [16]. Although, the specific amount of damage done to protein material by hypochlorous acid depends ultimately on the ratio of the active oxygen intermediates produced and the moles of protein material present available to quench the free radical reaction [17]. Sodium hypochlorite has long been used in situations where denaturing proteins is desirable [18]. In this work we tested the hypothesis that low levels of hypochlorite can denature fungal allergenic materials commonly found in the environment.

We used only commercially available extracts and not allergens. Although this precludes the possibility of determining exactly which of the fungal allergens were being measured it does allow for a more stringent test of the denaturing power of the hypochlorite in that all of the natural elements that interfere with hypochlorite action are present. This data should be understood in the context that fungal extracts are highly variable in the content of allergens and all of the allergens recognized by human IgE antibodies are not known. In addition it should be recognized that fungal derived allergens are cross reactive even between differing genera [19]. Therefore, we chose to test the available fungal extracts commonly used for skin testing instead of the individual allergens such as Alt a1 produced in bacteria by recombinant technology. Additionally, specific sera used in the study were chosen because they had individually tested positive for specific IgE content to either *Alternaria *or *Cladosporium *before entry into the serum pool. Before they were used in the experiment, the serum pool was again tested for IgE specific for the particular allergen extract used in these experiments.

The results of treating *Alternaria *or *Cladosporium *extract with solutions of sodium hypochlorite are immediate, obvious, and dramatic. At concentrations greater than 100 mM the noticeable brown color of the extract is completely bleached away and at concentrations between 100 and 30 mM substantial bleaching is seen. This was confirmed by all 3 of the immunoassay methods employed for measuring antigen and allergen content (Figure 1).

Inhibition immunoassay has long been employed for the quantification of fungal antigens in house dust [20,21]. As anticipated the results in Figure 1 indicate that greater than 80% of the *Alternaria *material recognized by the rabbit antisera is destroyed by hypochlorite treatment at concentrations greater than 50 mM and that more than 70% of the available epitopes are removed at concentrations greater than 30 mM.

One of the overriding concerns when using enzyme immunoassay in the context of a strong denaturing agent like hypochlorite is the potential impact of the hypochlorite on the antibodies that comprise the assay itself. To address this concern both sodium thiosulfite and bovine serum albumin sufficient to neutralize the hypochlorite treatment were added to the denatured mixture after an initial 10 minute treatment. Although this meant that the hypochlorite had only a 10 minute time period to act on the allergen material, it did allow us to employee the analytical accuracy of enzyme immunoassay to provide better quantization than electrophoresis methods used in previous experiments [4,5]. To further assure that our methods gave a true reflection of the allergenic material denatured, a positive control was constructed whereby material treated with high concentrations of hypochlorite was assayed in the presence of untreated material. For the experiment to be valid, the positive control determination for remaining epitopes must at least equal the determination of remaining epitopes in the untreated material that had been added back to the positive control. In this manner, we were able to assure that the decreased values determined were not because of diminished performance of the assay.

Direct or sandwich immunoassay with monoclonal antibodies is also frequently used to quantify allergen levels in house dust;[22] however, when using polyclonal antibodies these assays are susceptible to dramatic overestimations of the antigen present because of the variable nature of the house dust matrix. In the present case, this is not a concern because the matrix is held constant with respect to foreign antigens. The results of the direct immunoassay determination of viable *Alternaria *epitopes remaining after treatment with sodium hypochlorite is presented in Figure 1. In results similar to those determined with the inhibition immunoassay it is seen that at concentrations greater than 50 mM essentially 100% of epitopes are destroyed. This sandwich type of assay is expected see a greater amount of denaturing in that it requires 2 intact native epitopes on the same antigen molecule to produce a positive signal.

As in the results for the sandwich type assay using polyclonal rabbit sera raised against *Alternaria*, the sandwich immunoassay using human IgE from *Alternaria *allergic individuals in top layer of the assay is also less likely to recognize partially denatured *Alternaria *extract not only because 2 intact epitopes are required but also because at least one intact allergen epitope is also necessary. Figure 1 indicates that more than 40% of *Alternaria *allergen epitopes are destroyed at hypochlorite concentrations less than 10 mM.

The results for *Cladosporium *extract treated with similar concentrations of hypochlorite (Figure 2) are concordant with those from *Alternaria *extract. All treatments gave essentially 100% destruction of viable recognized epitopes at concentrations of hypochlorite greater than 50 mM and greater than 50% destruction at concentrations greater than 38 mM. The *Cladosporium *extract seemed to be slightly more resistant to the effects of hypochlorite than the *Alternaria *extract in that more epitopes survived at the lower treatment concentrations. There could be several reasons for this including that the *Cladosporium *extract may contain proportionately less protein material and therefore less allergen protein per gram of dry weight. Additional nonprotein and nonantigenic material in the mixture will mitigate the impact of the hypochlorite.

The reduction in the impact of hypochlorite in the presence of additional materials is a well recognized phenomenon; however, it did not prevent the results of the in vivo treatment of these 2 fungi growing on drywall material (Figure 3) from being very similar to the in vitro tests. When sprayed with hypochlorite concentrations of 88 and 38 mM, epitope destruction was in the 70 to 100% range for both *Alternaria *and *Cladosporium*.

Many previous authors have commented on the ability of hypochlorite solutions to denature allergenic materials [2-7]. In studies with the fungus *Aspergillus*[7] it was demonstrated that application of sodium hypochlorite onto mold-contaminated building material not only killed *A. fumigatus *but also reduced recognition of *A. fumigatus *mold extract by antibodies. Treatment with sodium hypochlorite also caused allergic extract materials derived from *Aspergillus *to lose the ability to produce positive skin tests in sensitized individuals. Work with recombinant rFel d1 [4] protein demonstrated that at a molar ratio of 560/1 hypochlorite/protein, the recombinant allergen was no longer recognized by monoclonal antibodies directed against it. Similarly the ability of rFel d1 to elicit histamine release from basophils of a cat allergic human was significantly reduced at a molar ration of 70/1 hypochlorite/protein [4]. It has been recently reported that Danish school children who lived in homes regularly cleaned with bleach had a lower incidence of asthma and eczema than their classmates [1]. In addition, the ability of regular cleaning using a combination of products containing hypochlorite, to produce an improvement in the quality of life of asthmatic children and their parents has been reported [2]. In an additional test of dilute hypochlorite in homes of asthmatic individuals researchers demonstrated significant reductions in the sum of all allergens measured before and immediately after cleaning and reductions in measured allergen levels over time. These reductions resulted in statistically significant improvements in asthma symptom parameters by questionnaire including wheeze, stuffy nose, itchy eyes, sneezing, and use of controller medicine [3].

## Conclusion

This work confirms the ability of solutions of sodium hypochlorite to denature fungal allergenic material from the common outdoor and indoor fungi *A. alternata *and *C. herbarum*. This destruction of recognized antigenic and allergenic epitopes occurs at concentrations of hypochlorite commonly used for household cleaning. This destruction of fugally derived allergenic material was demonstrated both in vivo and in vitro. This and other studies are necessary for the scientific understanding of the role of cleaning and environmental allergen removal in the allergic process and the alleviation of human symptoms related to allergic disease.

## End Note

Received support in part by Clorox Corporation.
